# Efficacy and safety of bismuth quadruple regimens containing tetracycline or furazolidone for initial eradication of *Helicobacter pylori*

**DOI:** 10.1097/MD.0000000000028323

**Published:** 2021-12-23

**Authors:** Junxian Wang, Yuping Cao, Wei He, Xiaoping Li

**Affiliations:** Second People's Hospital of Anhui Province, Hefei City, China.

**Keywords:** adverse event, furazolidone, *Helicobacter pylori*, quadruple therapy, tetracycline

## Abstract

*Helicobacter pylori* (*H pylori)* infection can cause chronic gastritis, peptic ulcer, and even gastric cancer, so effective eradication is critical.

This study compared the efficacy and safety of bismuth quadruple regimens including either tetracycline or furazolidone for initial eradication.

Patients newly diagnosed with *H pylori* infection from January 2020 to January 2021 were randomly assigned to receive either the tetracycline-containing regimen (n = 116) or furazolidone-containing regimen (n = 168). Both regimens included 1 proton pump inhibitor (rabeprazole 20 mg, or esomeprazole 20 mg, or eprazole 5 mg), colloidal pectin bismuth 300 mg, and amoxicillin 1000 mg in addition to tetracycline 1.0 g or furazolidone 0.1 g. All drugs were administered twice daily for 12 consecutive days. The ^14^C urea breath test was used for diagnosis, and re-test negativity at one-month follow-up was considered successful eradication. Adverse events were recorded during follow-up by telephone interview.

In total, 109 patients in the tetracycline group and 157 in the furazolidone group were re-examined at 1 month. In the tetracycline group, 101 patients tested negative at follow-up, yielding an eradication rate of 92.7% according to per-protocol analysis and 87.1% by intention-to-treat analysis. In the furazolidone group, 141 patients tested negative, yielding eradication rates of 89.8% by PP and 83.9% by ITT. Eradication rates did not differ significantly between regimens (per-protocol: *χ*^*2*^ = 0.637, *P* = .517; intention-to-treat: *χ*^*2*^ = 0.537, *P* = .501). However, total adverse events incidence was significantly lower in the tetracycline group (20.2% vs 37.6%; *χ*^*2*^ = 9.193, *P* = .003).

Both bismuth quadruple regimens produce high initial eradication, but the tetracycline regimen appears safer.

## 1 Introduction

*Helicobacter pylori* is a highly transmissible gastrointestinal pathogen detected in a large proportion of the global population. Rates of infection are particularly high in China,^[1]^ and although most cases are asymptomatic or result in mild gastritis, chronic *H pylori* infection is a major cause of peptic ulcer,^[2]^ and gastric mucosa-associated lymphoma^[3]^ and gastric cancer.^[4]^ In addition, *H pylori* infection is closely associated with the incidence of refractory iron deficiency anemia and idiopathic thrombocytopenic purpura (ITP). Eradication of *H pylori* can effectively reduce the risk of gastric cancer, so eradication therapy is now recommended for all positive patients according to the Kyoto Global Consensus Report on *H pylori* Gastritis.^[5]^ At present, the following treatment regimens for *H. pylori* eradication are commonly adopted in China: 2 antibacterial drugs + proton pump inhibitor + bismuth. Among them, the antibacterial drugs mainly consist of amoxicillin, tetracycline, clarithromycin, furazolidone, metronidazole and levofloxacin, and so on. Due to widespread application of antibacterial drugs, however, strains resistant to clarithromycin, levofloxacin, and metronidazole have emerged. At present, antibiotics with relatively low antibiotic resistance rates, including amoxicillin, furazolidone, and tetracycline, are recommended as part of multidrug regimens such as the bismuth quadruple regimen for the eradication of *H pylori*.^[6]^ Eradication and adverse event rates vary markedly among treatments and studies. In our preliminary clinical study, the adverse events incidence for quadruple bismuth regimens containing tetracycline or furazolidone exceeded 30%, significantly higher than previously reported.^[7,8]^ Therefore, the present clinical trial was designed to assess the efficacy and safety of bismuth quadruple regimens containing tetracycline or furazolidone for the initial eradication of *H pylori* among a relatively large cohort of newly positive patients at a single center.

## 2 Materials and methods

### 2.1 Study subjects

Patients diagnosed with initial *H pylori* infection as confirmed by ^14^C or ^13^C urea breath test^[9]^ from January 2020 to January 2021 and receiving no other treatment were randomly assigned to receive the same bismuth quadruple regimen but including either tetracycline or furazolidone. Exclusion criteria were as follows: allergic history to any drug in the quadruple regimen; malignancy confirmed by gastroscopy or moderate to severe gastric mucosal dysplasia indicated by pathological examination; >65 years or <25 years’ old; present pregnancy or lactation; history of gastric, esophageal, or duodenal surgery; receiving other antibacterial drugs or proton pump inhibitors (PPIs) within 1 month before treatment; severe primary diseases such as heart, lung, kidney, and brain disorders that may affect treatment; mental illness; failure to comply with treatment; long-term intake of non-steroidal anti-inflammatory analgesics or glucocorticoids; planning a pregnancy in the near future; and a history of *H pylori* eradication. In addition, eradication treatment was terminated for patients developing severe adverse reactions or exhibiting new symptoms of other diseases that may influence treatment response. All enrolled patients were informed of the clinical importance of *H pylori* eradication, possible failure rate, potential drug-induced adverse events, and the necessity of timely re-examination, and thereafter provided written consent. The study procedures were approved by the ethics committee of the Second People's Hospital of Anhui Province, a tertiary level hospital (No.2019-1104).

**Figure 1:**
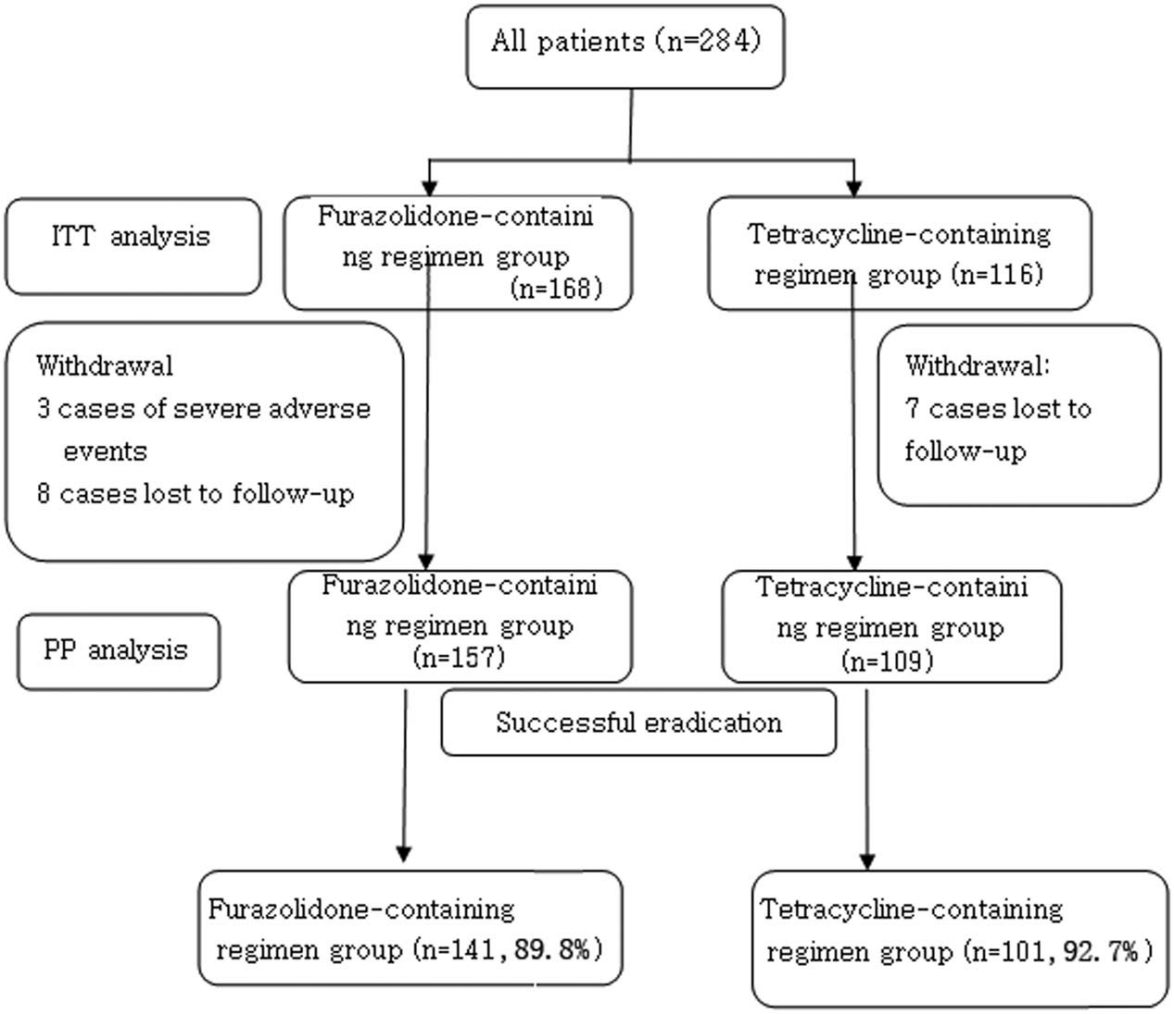
Flow chart of patient enrollment. Eradication rate was calculated according to both per protocol (PP) and intention-to-treat (ITT) principles.

Of the 284 patients allocated to receive one of the bismuth quadruple regimens, 15 were lost to follow-up, and the remaining 269 were monitored during treatment. Among these 269 patients, 3 withdrew due to severe adverse events and did not receive a second ^14^C or ^13^C urea breath test at 1 month, whereas the remaining 266 patients received re-examination and were included in efficacy and safety analyses. Figure 1 summarizes patient recruitment, group allocation, and follow-up, and Table 1 summarizes patient demographics. Neither mean age nor sex ratio differed significantly between tetracycline and furazolidone treatment groups.

**Table 1:** Patient demographics.

	Group		
Baseline data	Furazolidone-containing regimen group	Tetracycline-containing regimen group	*P*
No. of cases (n)	168	116	
Mean age, y	45.1 ± 10.7	43.04 ± 11.35	.114
Sex (male/female)	77/91	58/58	.546

### 2.2 Materials

In addition to twice daily oral tetracycline or furazolidone, the eradication regime included twice daily oral rabeprazole 20 mg, (or esomeprazole 20 mg, or eprazole 5 mg), amoxicillin 1000 mg, and colloidal pectin bismuth 300 mg. All drugs were prescribed for 12 consecutive days. Infection was diagnosed and re-examined at follow-up using a Headway HUBP-01 ^14^Cor ^13^C breath detector (Shenzhen Zhonghe Headway Bio-Sci & Tech Co., Ltd.).

### 2.3 Methods

#### 2.3.1 Drug administration and follow-up

The PPI (rabeprazole,or esomeprazole, or eprazole) and colloidal pectin bismuth was taken 30 minutes before meals, whereas amoxicillin and tetracycline or furazolidone were taken after meals. Telephone follow-up interviews were conducted during and following the drug regimen to document adverse events.

#### 2.3.2 Assessment of infection and eradication efficacy

Patients were diagnosed according to a disintegrations per minute (dpm) ≥100 on the ^14^C urea breath test or dob ≥4 on the ^13^C urea breath test. A reading of dpm <100on the ^14^C urea breath test or dob <4 on the ^13^C urea breath test at 1 month post-treatment was defined as successful eradication of *H pylori*. The testing procedures were performed as previously described.^[10]^

#### 2.3.3 Per-protocol analysis

The enrolled patients who completed the treatment plan statistically analyzed to evaluate the effectiveness of corresponding treatment.

Intent-to-treat (ITT) analysis: The patients who were eligible for the treatment regimens, randomly divided into different groups and began to receive corresponding treatment (including the evaluation of cases lost to follow-up) were statistically analyzed to evaluate the overall effectiveness. Both per-protocol (PP) and ITT analyses were commonly employed to objectively evaluate the effectiveness of *H pylori* eradication.

### 2.4 Statistical analysis

All results were analyzed using SPSS16.0 (SPSS Inc., Chicago, IL). Demographic parameters were compared between treatment groups by unpaired *t* test or *χ*^*2*^ test. Eradication rate and adverse events incidence were also compared by *χ*^*2*^ test. A *P* < .05 (2-tailed) was considered statistically significant for all tests.

## 3 Results

### 3.1 Eradication efficacy

Of the 284 cases enrolled and allocated to one of the treatment groups, 15 were lost to follow-up and 3 withdrew because of serious adverse events (1 case of high fever complicated with diarrhea, 1 case of extensive rash, and 1 case of recurrent nausea and vomiting). Finally, 266 patients completed treatment and follow-up, including re-examination by ^14^C urea breath test (93.7%) (Fig. 1). Among 266 patients, 242 cases received successful eradication and the remaining 24 cases experience eradication failure. Of the 109 cases remaining in the tetracycline-containing group, 101 tested negative for *H pylori* at 1 month, for an eradication rate of 92.7% according to PP analysis and 87.1% by ITT analysis. Of the 157 cases remaining in the furazolidone group, 141 tested negative, for an eradication rate of 89.8% by PP analysis and 83.9% by ITT analysis. Eradication rate did not differ significantly between 2 groups according to PP analysis (*χ*^*2*^ = 0.637, *P* = .517) or ITT analysis (*χ*^*2*^ = 0.537, *P* = .501) (Table 2).

**Table 2:** Comparison of *Helicobacter pylori* eradiation rates (%) between regimes.

	Group		
Sample	Furazolidone-containing regimen	Tetracycline-containing regimen	*P*
ITT	83.9	87.1	.501
PP	89.8	92.7	.517

ITT = intention-to-treat, PP = per-protocol.

### 3.2 Incidence of adverse events

Telephone follow-up interviews were conducted with all 269 patients during the regimen to provide guidance on drug intake, improve treatment compliance, and record adverse reactions. The most common adverse events were nausea, decreased appetite, metallic taste in the mouth, dizziness, fatigue, hand and foot numbness, insomnia, diarrhea, rash, and fever. Black stool was also reported but not considered an adverse event. Three patients in the furazolidone-containing regimen group withdrew due to serious adverse reactions (1 case of high fever complicated with diarrhea, 1 severe vomiting and 1 severe rash), and all these symptoms were mitigated by active symptomatic treatment. One patient complained of tetania during treatment but no abnormality was found by hospital examination. One patient also complained of palpitation during the regimen but no arrhythmia by detected by ECG. Three cases complained of transient hand and foot numbness after taking the medication, whereas other adverse reactions were tolerable, and all three completed the regimen without special interventions. Treatment compliance was relatively high because all patients were informed of potential adverse reactions before treatment. The total incidence of adverse events was significantly lower in the tetracycline-containing regimen group than the furazolidone-containing regimen group, which included all three cases of severe adverse events (*χ*^*2*^ = 9.199, *P* = .003) (Table 3).

**Table 3:** Comparisons of individual adverse events (n) between groups.

Adverse event	Furazolidone-containing regimen group	Tetracycline-containing regimen group	*P*
Total no. of cases	59	22	.003
Vomiting	4	1	
Nausea	16	5	
Lack of appetite	21	14	
Abdominal pain	4	5	
Diarrhea	6	4	
Fever	11	0	
Fatigue	19	13	
Rash	2	0	
Parageusia	26	9	
Dizziness	6	5	
Insomnia	9	3	
Tetania	1	0	
Palpitation	1	0	
Hand and foot numbness	3	0	

## 4 Discussion

Both the furazolidone-containing and the tetracycline-containing bismuth quadruple regimens yielded eradication rates for initial *H pylori* infection ≥80%, and thus are acceptable treatments according to the Maastricht III Consensus Report. Furthermore, both regimens were tolerated by the majority of patients. However, adverse reaction rates were high for both regimens,^[11]^ particularly for the furazolidone-containing regimen. Based on these findings, the tetracycline-containing regimen may be preferable as first-line treatment.

Treatment for *H pylori* eradication may fail for a variety of reasons, but antibiotic resistance is regarded as the main cause.^[12]^ Amoxicillin can eliminate *H pylori* directly with low antibiotic resistance. In addition, it has been reported that amoxicillin can protect the gastrointestinal mucosa from injury by increasing local blood flow and prostaglandin E release. Further, amoxicillin evokes few adverse reactions, and so is included in most regimens for eradicating *H pylori*.

Furazolidone is a nitrofuran antibiotic that damages bacterial DNA and interferes with normal bacterial metabolism. Studies have demonstrated that furazolidone can also provide a degree of protection to the gastric mucosa.^[7]^ Indeed, furazolidone was widely used to treat duodenal ulcer in China even before it was recommended for *H pylori* eradication.^[13]^*H pylori* also demonstrates low primary and secondary resistance to furazolidone, and no cross-resistance with metronidazole. In the present study, dosage (100 mg twice daily) was set according to national consensus guidelines. A *Meta*-analysis reported that high-dose furazolidone yielded greater *H pylori* eradication than low-dose furazolidone (100 mg PO bid), but also significantly increased the risk of adverse reactions.^[14]^ However, another study found no difference in eradication rate between high- and low-dose furazolidone, but noted markedly higher adverse response rate to high-dose furazolidone.^[15]^ In the present study, the recommended dose was associated with higher adverse event frequencies than reported previously, especially parageusia, poor appetite, nausea, and fever. Fever was reported approximately 1 week after the start use of the medication regimen, but was alleviated in all cases by non-steroidal antipyretic analgesics. Furthermore, fever symptoms were not worsened by continued use of furazolidone, and only 1 patient withdrew due to severe fever. Therefore, we believe that furazolidone can be administered to cycle completion even if fever emerges. However, careful monitoring for fever and other adverse events is critical during the early stage of the regimen.

Tetracycline is a broad-spectrum but relatively low-potency antibiotic produced by actinomycetes. *H pylori* has low resistance to tetracycline, so it is recommended as a first-line drug for *H pylori* infection in many countries, including China and the United States.^[8]^ Tetracycline suppresses bacterial reproduction and viability by interfering with protein synthesis. In contrast to tetracycline, amoxicillin is most effective against actively reproducing bacteria, suggesting that these two agents may be complementary or act synergistically. However, the efficacy of this combination remains controversial.^[16,17]^ In the present study, combined high-dose tetracycline (1 g PO bid) and amoxicillin yielded high efficacy *H pylori* eradication, with lower total adverse events frequency than the furazolidone-containing regimen. Nonetheless, individual adverse event rates were relatively high, especially loss of appetite. Furthermore, tetracycline can cause severe liver damage,^[18]^ although no early signs of liver dysfunction were detected in this study, including among patients with fatty liver disease. In addition to liver damage, tetracycline can affect the growth of teeth and bone.

Bismuth is used for eradicating *H pylori* due to its capacity for directly destroying bacterial cell membranes and suppressing the production of essential metabolic enzymes and cytoprotective proteins such as urease, phospholipase, heat shock protein, and histidine-rich protein, which in turn can induce bacterial oxidative stress and apoptosis. Through these actions, bismuth can enhance the sensitivity of bacteria to other antibiotics, allowing lower doses for greater safety.^[19]^ Therefore, bismuth is included as a component of the standard quadruple regime in Chinese guidelines for eradication of *H pylori*. In our clinical practice, no severe adverse reactions inducted by bismuth were observed.

Inclusion of a PPI can improve the efficacy and safety of regimens for eradicating *H pylori* by promoting antibiotic stability and increasing the minimum inhibitory concentration.^[20]^ However, the recommended dosages vary widely. Yong et al^[21]^ found no significant difference in *H pylori* eradication rate between regimens including 10 or 20 mg PO bid rabeprazole, but others have found that increasing the PPI dosage can improve the eradication rate.^[22]^ In the present study, a relatively large PPI dose was thus adopted. Individual patient sensitivity to PPIs such as omeprazole and lansoprazole may vary due to allelic polymorphism of the *CYP2C19* gene.^[23]^ In China, approximately 50% of the population demonstrates rapid PPI metabolism by the *CYP2C19* gene.^[24]^ To reduce the potential impact of *CYP2C19* gene polymorphisms on *H pylori* eradication, we chose PPIs (rabeprazole, eprazole, and esmomeprazole) known to be less sensitive to *CYP2C19* genotype.^[25]^ Previous studies have demonstrated all three PPIs can promote high eradication efficacy when combined with the appropriate antibiotics through rationale medicine. Consequently, we recommend PPIs that are less affected by *CYP2C19* gene polymorphisms conferring a higher rate of liver metabolism. Newly developed PPIs such as vonoprazan^[26]^ may also yield high efficacy eradication of *H pylori* due to potent acid inhibition effects and thus warrant additional clinical investigation.

This study has several limitations. The efficacy of *H pylori* eradication can be influenced by multiple factors in addition to drug combination and dose,^[27]^ including the type and stage of *H pylori* infection-related disease,^[28]^ patient age, sex, and treatment compliance,^[29]^ smoking, drinking, gastric acid environment, the load of *H pylori*, the specific infecting strain, transformation of *H pylori* into the coccoid form,^[30]^ colonization site, and bacterial internalization,^[30]^ and none of these factors was considered. In addition, individual adverse events associated with specific drugs were not analyzed due to the limited sample numbers. Hence, this study can provide no guidance for selecting specific drugs with best adverse events profiles for individual patients. In subsequent larger-scale clinical trials, these various factors should be considered for effects on first *H pylori* eradication and subsequent treatment for recurrence. Also, the antibiotic resistance of *H pylori* to each drug in the regimen should be analyzed. Additional studies on the impacts of bacterial internalization, transformation into the coccoid form, mutations of antibiotic resistance genes, and host CYP2C19 genotype are needed to identify the safest and most efficacious drug combinations for *H pylori* eradication.

Taken together, quadruple regimens containing tetracycline or furazolidone yield a high eradication rate of *H pylori.* Although these 2 quadruple regimens show high incidence of adverse reactions, these symptoms are primarily mild, and most of them do not need to be treated. Therefore, these 2 regimens can be adopted as the primary option for eradication of *H.pylori.* However, clinicians should fully explain the potential risk of adverse reactions to the patients and improve their levels of compliance.

## Acknowledgment

The authors appreciate the assistance from Endoscopic Center of Anhui No.2 Provincial People's Hospital.

## Author contributions

Wang JX and Cao YP contributed study concept, design, analysis and interpretation of data; Cao YP, He W, Li XP contributed acquisition of data; Wang JX and Cao YP wrote the manuscript; All authors wrote, read and approved the final manuscript.

**Conceptualization:** Yuping Cao, Wei He.

**Data curation:** Yuping Cao, Xiaoping Li.

**Writing – original draft:** Junxian Wang.

**Writing – review & editing:** Junxian Wang.
